# Interactions between Intestinal Microbiota and Host Immune Response in Inflammatory Bowel Disease

**DOI:** 10.3389/fimmu.2017.00942

**Published:** 2017-08-14

**Authors:** Ming Zhang, Kaiji Sun, Yujun Wu, Ying Yang, Patrick Tso, Zhenlong Wu

**Affiliations:** ^1^State Key Laboratory of Animal Nutrition, Department of Animal Nutrition and Feed Science, China Agricultural University, Beijing, China; ^2^Department of Pathology and Laboratory Medicine, Metabolic Diseases Institute, University of Cincinnati, Cincinnati, OH, United States; ^3^Beijing Advanced Innovation Center for Food Nutrition and Human Health, China Agricultural University, Beijing, China

**Keywords:** intestinal microbiota, host immune response, inflammatory bowel disease, intestinal barrier function, epithelial cells

## Abstract

Inflammatory bowel disease (IBD) is a chronic inflammatory disorder of the gastrointestinal tract. Although the etiology and pathogenesis of IBD remain unclear, both genetic susceptibility and environmental factors are implicated in the initiation and progression of IBD. Recent studies with experimental animal models and clinical patients indicated that the intestinal microbiota is one of the critical environmental factors that influence nutrient metabolism, immune responses, and the health of the host in various intestinal diseases, including ulcerative colitis and Crohn’s disease. The objective of this review is to highlight the crosstalk between gut microbiota and host immune response and the contribution of this interaction to the pathogenesis of IBD. In addition, potential therapeutic strategies targeting the intestinal micro-ecosystem in IBD are discussed.

## Introduction

Inflammatory bowel disease (IBD), including ulcerative colitis (UC) and Crohn’s disease (CD), is a chronic and relapsing inflammatory condition of the gastrointestinal (GI) tract characterized by abdominal pain, diarrhea, and bloody stools (1–3). IBD affects approximately 3.7 million people in North America and Europe (4–6). Furthermore, an increasing incidence of IBD has been observed in Asian countries, such as China (7), India, South Korea (4, 8), and Saudi Arabia (9), over the past two decades. Although CD and UC share partially overlapping pathological and clinical symptoms, there are distinct clinical characteristics; for example, CD can affect one or several segments of the digestive tract, whereas UC is mainly restricted to the mucosal layer of the colon or rectum without affecting other areas of the GI tract (2, 10). The clinical manifestations of IBD have been well-defined many years ago. Nevertheless, the etiologies and pathogenesis of this disorder remain largely elusive (11). The identification of genetic loci associated with susceptibility to IBD has indicated the role of genetic background in the pathophysiology of IBD (12). However, genetic polymorphism alone does not predict the development of IBD, thus highlighting the involvement of other environmental factors in pathogenesis (13).

The human gut is the habitat for 100 trillion of different microbial organisms, including bacteria, viruses, fungi, and protozoans, which are 10 times more than all of the cells in the human body (14, 15). It is generally believed that the gut of human is colonized by commensal microbes from the vaginal canal, skin, feces, or breast milk during delivery or after birth (16, 17). The phylogenetic diversity of the intestinal microbiota increases with growth and development and ultimately leads to a complex and relatively stable community of microorganisms at the age of roughly 2–3 years (18, 19). Based on culture-independent molecular profiling methods (20, 21), it is estimated that more than 1,000 species of bacteria are represented in the GI tract, and the majority of them are obligate anaerobic organisms, including *Firmicutes, Bacteroidetes, Proteobacteria*, and *Actinobacteria* (22). Among them, *Firmicutes* (Gram-positive) and *Bacteroidetes* (Gram-negative) are the predominant phyla across all mammalian species, accounting for over 90% of all intestinal bacteria (12, 23–25). The total number and composition of bacteria vary in different segments of the GI tract, with a relatively low number and few species of bacteria residing in the stomach and upper part of the small intestine. The number of resident microbes increases from the jejunum to each subsequent part of the gut, reaching up to 10^12^/g in the feces (18, 26).

Changes in the composition of intestinal bacteria could influence intestinal homeostasis through various signaling pathways, thus affecting the interactions between bacteria and the host (27, 28). Intestinal bacteria are responsible for the degradation of indigestible carbohydrates to produce short-chain fatty acids (SCFAs), synthesis of vitamins (vitamin K, vitamin B12, and folic acid), synthesis of amino acids, and regulation of fat metabolism, which are required for the integrity of intestinal barrier function (29–31). Accordingly, studies have observed that the small intestinal crypts of germ-free mice are atrophic and have a decreased proliferation rate and impaired angiogenesis (32, 33). In addition, the bacteria in the gut protect the intestinal epithelium from the harmful effects of pathogens by inhibiting the colonization of pathogenic bacteria and producing antimicrobial compounds, thus contributing to homeostasis (34). Furthermore, the intestinal microbiota actively stimulates and promotes the development and maturation of the immune system, which is required for the body’s defense against pathogens and peripheral immune tolerance against potential antigens in the lumen (35–37). The resident intestinal microbiota is vital for the physiology and health of the host. However, this does not mean that a particular species is entirely beneficial (38). *Bacteroides fragilis*, a commensal bacterium that is well controlled under normal conditions, has been reported to invade intestinal tissues and pose a serious threat in immunocompromised individuals (39).

The diversity and composition of the intestinal flora can be altered by diet, environmental factors, stress, lifestyle, exogenous probiotics, and antibiotic use (40, 41), which in turn would affect intestinal homeostasis. The mutual relationship and interaction between the intestinal flora and host have increasingly attracted the attention of both research scientists and clinical practitioners, and this has led to a greater understanding of the human microbiome in recent years (42). Consistently, the dysfunction of the interaction between the intestinal microbiota and host can lead to an excessive inflammatory response and contribute to the initiation and/or progression of IBD (28), intestinal bowel syndrome, and functional dyspepsia in humans (43), thus highlighting the critical role of the intestinal microbiota in health and IBD (44). Given these observations, IBD is regarded as an excessive activation of the host immune response to the intestinal microbiota in genetically susceptible patients (7). Herein, we summarize the current literature that describes the association between gut microbiota and host immune response in the pathogenesis of IBD as well as the potential therapeutic strategies involving the modulation of the intestinal micro-ecosystem.

## Intestinal Microbiota in IBD

A large number of studies have shown that the disturbance of normal microbial populations in the GI tract is linked to both acute infections, such as *Clostridium difficile* infection (CDI) and to chronic diseases, including IBD, irritable bowel syndrome, metabolic diseases, and autoimmune disorders (45, 46). The term “dysbiosis” was first coined by Metchnikoff in the early twentieth century to describe changes in intestinal bacteria, which has been further defined and extended by others (47). The concept of dysbiosis implies that an imbalance in the microbial ecosystem disrupts immune homeostasis and leads to intestinal disorders, including both CD and UC (48). It emphasizes the critical role of the interactions between the intestinal flora and host immune system in the pathogenesis of intestinal diseases (49). In an initial study conducted in 2002, Swidsinski et al. demonstrated that the abundance of mucosal microbiota in patients affected with IBD is positively correlated with disease severity (50). Although they did not observe substantial changes in the composition of the intestinal flora in IBD patients because of technological limitations (24, 25), this is the first study showing a possible relationship between bacteria and IBD. In the last decade, the application of culture-independent molecular approaches in the study of intestinal microbiota diversity has improved our understanding of the intestinal microbiota and immune response in intestinal disorders (51). Metagenomic studies demonstrated that microbial diversity and intestinal microbiota stability decrease in IBD patients compared with individuals without IBD (52). Consistently, 25% fewer genes were detected in the fecal samples of IBD patients than in those of control patients (26). Further studies have shown that IBD patients have fewer bacteria with anti-inflammatory properties (bacteria in phyla *Firmicutes*) and/or more bacteria with pro-inflammatory properties (24, 53–55). Joossens et al. demonstrated that the fecal microbiota in CD patients has a reduced abundance of anti-inflammatory *F. prausnitzii, B. adolescentis*, and *D. invisus* and an increased abundance of potentially pro-inflammatory *R. gnavus* (56). This change in the intestinal flora might contribute to chronic inflammation as observed in the GI tract of IBD patients.

Another bacterium with pro-inflammatory properties is *E. coli* AIEC (adherent/invasive *E. coli*), a mucosa-associated *E. coli* with strong adhesive–invasive properties, which was originally isolated from adult CD patients (57). In comparison with normal controls and patients with colonic CD, about 38% of patients with active ileal CD have been found to have an increased concentration of AIEC (58, 59). *Campylobacter concisu*s, another invasive proteobacterium, has also been reported to preferentially colonize CD patients (60–62). The increase in pathogenic bacteria that adhere to intestinal epithelial cells affects intestinal permeability, alters the diversity and composition of gut microbiota, and induces inflammatory responses by regulating pro-inflammatory gene expression, ultimately resulting in colitis (63). In addition, the disruption of intestinal bacteria affects the pathogenesis of IBD by their metabolites. For example, the SCFAs produced by commensal bacteria exert anti-inflammatory activity and serve as a major energy source for the colonic epithelium. The production of SCFAs has been reported to decrease in IBD-affected patients because of decreased *F. prausnitzii*, a butyrate-producing bacterium in the gut (64–67). By contrast, the concentration of sulfate-reducing bacteria is higher in IBD patients, which can result in the metabolic production of hydrogen sulfide that is toxic to intestinal epithelial cells and induce mucosal inflammation, thus leading to UC (68). Taken together, the overall data have indicated a strong correlation between intestinal microbiota alteration and the pathogenesis of IBD (69–71). However, it should be kept in mind that the diversity and composition of bacteria among experimental animal studies or IBD patients are not consistent, and the results are contradictory under some conditions (72). Moreover, it remains unclear whether the observed changes in phylogenetic composition are causative for the development of IBD or simply a consequence of an altered intestinal environment during the progression of IBD (18, 72, 73). Additional studies and an in-depth analysis of the gut flora are needed to address these issues before any conclusions can be drawn.

## Immune Mechanisms for Intestinal Homeostasis Maintenance

**Figure 1:**
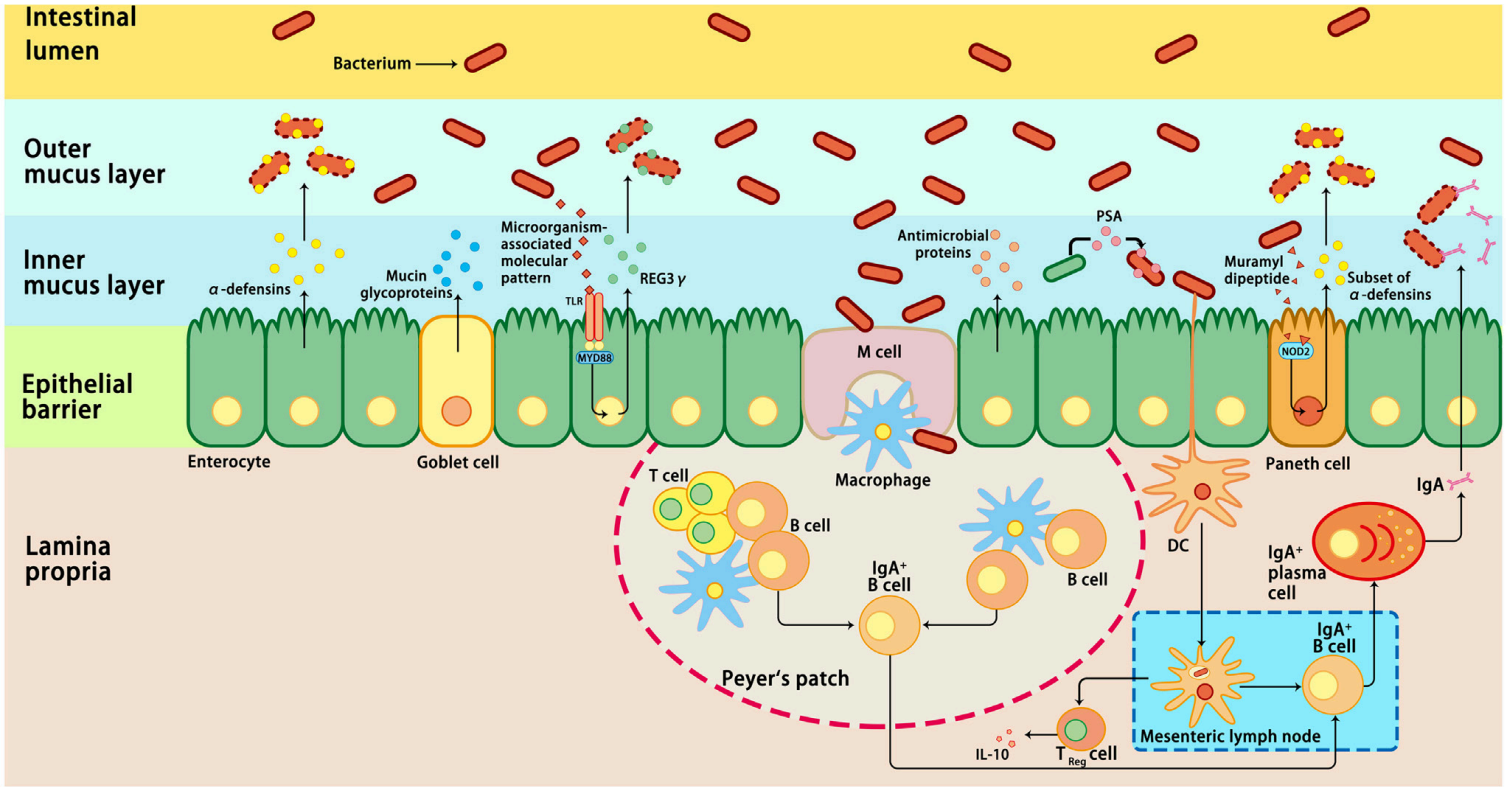
Host immune responses to intestinal microbiota. Several immune mechanisms work in concert to the intestinal microbiota and contribute to intestinal homeostasis. Goblet cells secret mucin glycoproteins, plasma cells secret IgA, and epithelial cells secrete antimicrobial proteins through toll-like receptors (TLRs), or nucleotide-binding oligomerization domain-containing protein 2 (NOD2)-dependent mechanisms. Dendritic cells (DCs) take up bacteria migrate to Peyer’s patches and mesenteric lymph nodes where B cells are differentiated into IgA-secreting plasma cells. In addition, sampling of polysaccharide A (PSA) from *Bacteroides fragilis* by intestinal DCs leads to induction of regulatory T (Treg), which is responsible for the production of IL-10. In addition, the antimicrobial proteins secreted by the host cells can modulate the composition of the microbiota. IL-10, interleukin 10; M cell, microfold cell; MYD88, myeloid differentiation primary-response protein 88; REG3γ, C-type lectin regenerating islet-derived protein 3γ.

Intestinal epithelial cells are in direct contact with a variety of xenobiotic factors, including pathogenic microorganisms, dietary antigens, or toxic components, which can trigger and activate the immune system of the host (74, 75). Inflammation is a protective response of the immune system to infection or tissue injury. However, prolonged or chronic inflammation is detrimental and associated with the development of IBD (76). Therefore, an appropriate immune response to intestinal pathogens without eliciting an inflammatory response to commensal bacteria is critical for the maintenance of intestinal homeostasis (77). Host immune cells have evolved various mechanisms to ensure intestinal homeostasis, including the mucosal and epithelial barrier, pro-inflammatory signaling pathways, and intestinal innate and adaptive components (78) (Figure 1). These mechanisms allow a relative stable bacterial population to form and limit the colonization of pathogenic bacteria and microbiota-driven inflammation (76, 79). First, the intestinal barrier formed by epithelial cells provides a physical barrier separating the luminal contents from the underlying immune compartments, thereby blocking the entry of microflora into the lamina propria (76). Second, the specialized secretory cells in the GI tract, such as plasma cells, goblet cells, and Paneth cells, produce and secret IgA, mucus, and antimicrobial proteins, respectively; these make up the main components of the intestinal mucosa, which function as a defense line to reduce the microbial burden of the epithelium (38). The expression of antimicrobial proteins in the gut is regulated by distinct mechanisms (Figure 1). For example, most α-defensins are constitutively expressed, whereas the expression of β-defensins, C-type lectin regenerating islet-derived protein 3γ, and a subset of α-defensins is regulated by bacteria-activated toll-like receptor or nucleotide-binding oligomerization domain-containing protein 2 (NOD2) signaling (80). Third, microbiome-derived metabolites or small molecules with microbicidal or microbiostatic properties promote resistance to colonization by pathogenic species. For instance, polysaccharide A, a microbial molecule synthesized by *B. fragilis*, has been reported to prevent colitis induced by *Helicobacter hepaticus* through suppression of interleukin (IL)-17 production and enhancement of IL-10-producing CD4^+^ T cells (81). Fourth, when commensal bacteria penetrate the intestinal epithelial cells, the innate and adaptive immunity systems can be activated to eliminate the microbiota (38). A combination of the above-mentioned actions in epithelial cells, secretory cells, and immune cells ensures a state of limited mucosal response to commensal bacteria (38, 76). Importantly, immune responses induced by commensal flora can regulate the composition of the intestinal microbiota, thus maintaining the dynamic balance between commensal bacteria and the host immune system and ensuring gut homeostasis and health (82).

## Dysregulated Interaction Between Intestinal Microbiota and Host Immune Response in IBD

**Figure 2:**
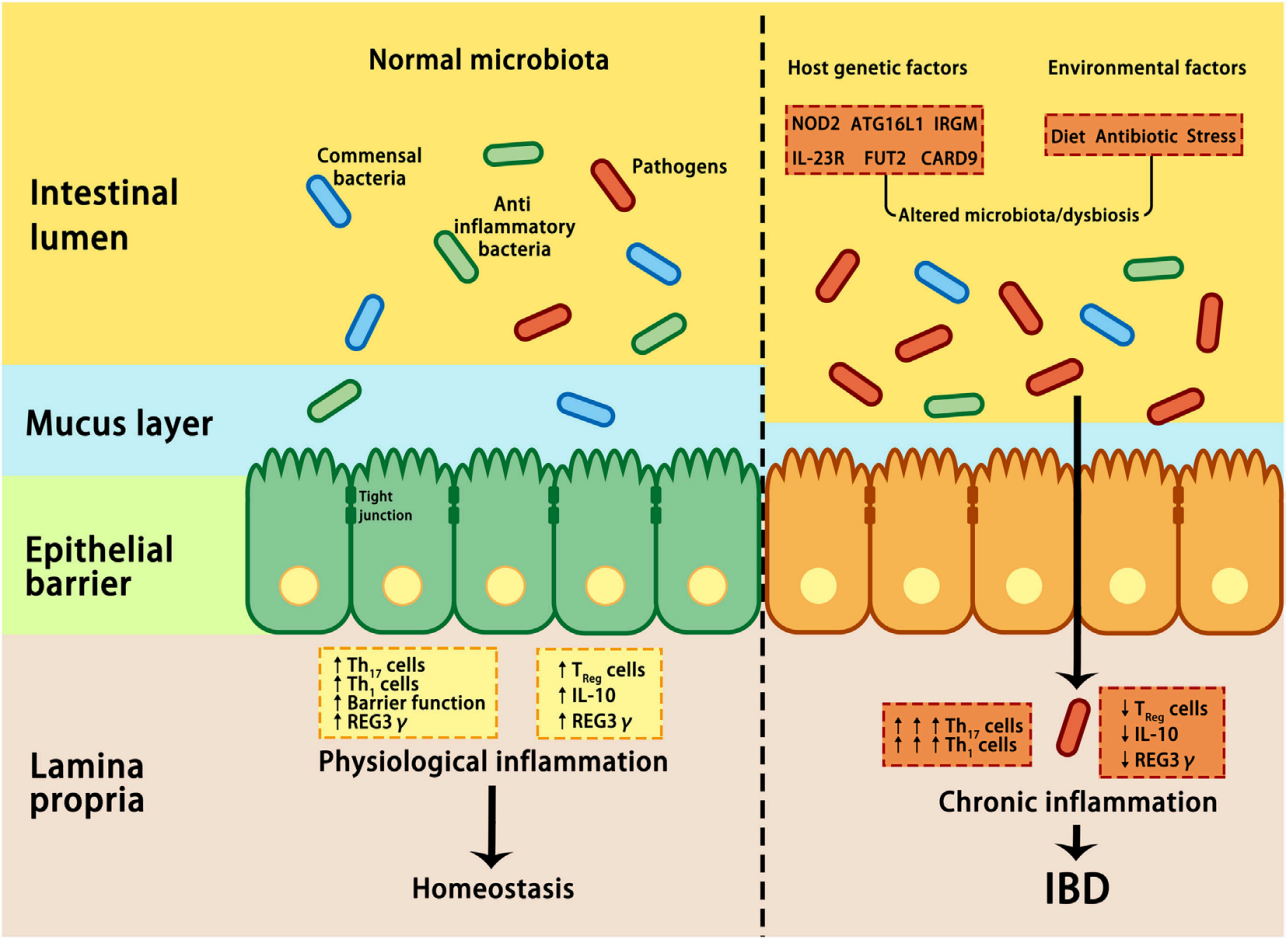
Interactions between microbiota and host genetic and environmental factors contribute to the pathogenesis of IBD. Under healthy conditions (left panel), pathogens are suppressed by beneficial commensal bacteria through the induction of antimicrobial proteins, such as IL-10 and REG3γ, thus maintaining homeostasis. In IBD (right panel), a combination of genetic factors and environmental factors (such as stress, diet, and antibiotic) lead to dysbiosis, which in turn affects barrier integrity, innate, and adaptive immunity, resulting in uncontrolled chronic inflammation and hyper-activation of T helper 1 (Th1) and Th17 cells, increase in tight junction permeability, reduction in regulatory T (Treg) cells, and decrease in REG3γ and IL-10. ATG16L, autophagy-related 16-like; CARD9, caspase recruitment domain family member 9; FUT2, fucosyltransferase 2; IBD, inflammatory bowel disease; IL-10, interleukin 10; IRGM, immunity-related GTPase M; NOD2, nucleotide-binding oligomerization domain-containing protein 2; REG3γ, C-type lectin regenerating islet-derived protein 3γ.

Despite the mucus, antimicrobial proteins and secretory IgA are critical components for maintaining intestinal homeostasis, several GI pathogens can penetrate the epithelial barrier and contribute to the pathogenesis of IBD if they are not eliminated through host immune reactions (38). By using non-aqueous Carnoy fixative, Swidsinski et al. demonstrated increased bacterial adherence to the mucosal surface of IBD patients, indicating a decreased ability to limit direct contact between the epithelium and intestinal microbiota and the potential over-activation of the host immune response (83) (Figure 2). Based on this observation, IBD is regarded as a chronic inflammatory disorder caused by an excessive immune response to intestinal flora. Many susceptibility genes and environmental factors have been identified to interfere with the interactions between the microbiota and host immune system through various signaling pathways in IBD patients (27, 28, 82, 84, 85).

### Genetic Factors Affecting the Interactions between Intestinal Microbiota and Host Immune Response in IBD

Nucleotide-binding oligomerization domain-containing protein 2 plays an important role in intestinal homeostasis by activating nuclear factor-κB or the mitogen-activated protein kinase (MAPK)-mediated immune response upon sensing specific bacterial peptidoglycan motifs in the cytosol (86). The activation of NOD2 signaling also enhances the production of antimicrobial peptides and mucin, two critical components of the intestinal mucosa that prevent the bacteria from colonizing epithelial cells (86). As a critical regulator of inflammation, mutations of NOD2 are associated with decreased *Clostridium* group XIVa and IV (SCFA-producing bacterial strains) and increased *Actinobacteria* and *Proteobacteria*, resulting in an intestinal flora shift in patients with CD and UC and thus leading to a higher susceptibility to inflammation (87–91). Consistently, NOD2-deficient mice have compromised epithelial barrier, reduced intraepithelial lymphocytes, decreased α-defensin production, and impaired immune responses to pathogenic bacteria, which are indicative of experimental colitis (92–94). In addition to NOD2, other IBD susceptibility genes, such as ATG16L1, XBP1, IRGM, CARD9, and FUT2, have been reported to affect innate and/or adaptive immune functions as well as the composition and diversity of the intestinal flora (95, 96). The ATG16L1 or XBP1 gene variants are associated with disturbances in Paneth and/or goblet cell function (97–99) and increased IL-1β production in response to bacterial peptidoglycan muramyl dipeptide (95), thus contributing to the ileal disease phenotype in CD. Recently, fucosyltransferase 2 (FUT2), a gene that regulates the expression of H antigens (precursors of blood group A and B antigens) in the GI mucosa, has been reported to be involved in regulating the structure and composition of the intestinal microbiota, thereby significantly contributing to CD susceptibility (100). Genome-wide association studies have identified over 163 single nucleotide polymorphisms (SNPs) associated with different susceptibilities to CD or UC, and more loci will no doubt be reported in the future (101–103). Most of the identified genes (110 of 163 gene loci) are shared by both CD and UC, whereas only 23 and 30 loci are specifically correlated with susceptibility to UC and CD, respectively (103, 104). These susceptibility genes primarily regulate host immune response signaling (101–103). Recent studies have shown that dysregulation of the IL-23/Th17 axis is associated with multiple genetic susceptibility SNPs in patients with CD and UC because of impairment in the innate and adaptive immune response (105, 106). Notably, intestinal dysbiosis is associated with the elevated generation of reactive oxygen species (107), which in turn can lead to changes in the composition and diversity of the intestinal microbiota (108), increased mucosal permeability (73), and increased immune stimulation, thus forming a vicious cycle. Nevertheless, more studies are required to elucidate the mechanism of the bidirectional regulation between the microbiota and host immune response under specific conditions (109, 110). An example of how specific microbes induce intestinal inflammation and influence the pathogenesis of IBD was reported by Bloom et al. (111). In their study, commensal *Bacteroides* species were isolated in IL-10r2- and Tgfbr2-deficient mice (111). Despite the colonization of the isolated bacteria in both IBD-susceptible and non-susceptible mice, IBD induction was exclusively observed in susceptible animals, thus providing an important insight into the role of intestinal dysbiosis in IBD induction (112). Considering that commensal *Bacteroides* species are present in abundance in the mammalian intestine and have beneficial effects on the host through the breakdown of complex dietary carbohydrates, modulation of mucosal glucosylation, and immune maturation (46), classically beneficial bacteria might have detrimental effects under specific conditions and could contribute to IBD (112). These data also support the critical role of genetic factors in the development of IBD *via* regulation of intestinal bacterial composition and diversity as well as immune responses in the GI tract. It should be kept in mind that the susceptibility alleles are not sufficient on their own to trigger IBD. The evidence thus far indicates that the development of IBD is a combined effect of both genetic and non-genetic factors, which act together and lead to changes in the structure and function of the human intestinal microbiota (113).

### Environmental Factors Affecting the Interactions between Intestinal Microbiota and Host Immune Response in IBD

The composition of the intestinal flora is affected by various environmental factors, such as diet, stress, age, and antibiotic treatment (114). In this section, we will mainly focus on diet and stress as well as their effects on the interactions between the intestinal microbiota and host immune response.

#### Diet

It is well known that dietary components affect the structure and activity of intestinal bacteria (115, 116). Western food intake (high sugar/fat and low dietary fibers, fruits, and vegetables) is associated with the altered structure and function of commensal flora in the gut (113, 117), which might favor an increased incidence of IBD (6, 8, 118). In a recent study, David et al. reported that the short-term (5 days) consumption of animal-based foods is associated with an increased abundance of bile-tolerant microorganisms (*Alistipes, Bilophila*, and *Bacteroides*) and reduced *Firmicutes* that metabolize dietary plant polysaccharides, demonstrating the rapid modulatory effect of dietary nutrients on intestinal bacteria (116). Considering that the production of H_2_S by *Bilophila wadsworthia*, a sulfite-reducing bacterium, might trigger inflammatory responses in the intestine (119), these data provide a plausible explanation for the higher prevalence of IBD in Western populations. Similar results were observed when switching from a plant-based diet to a typical Western diet for 1 day (115), thus highlighting the possibility of restoring the gut microbiota in IBD by nutritional interventions.

Dietary fibers can be converted to SCFAs by anaerobic bacteria (*Bacteroidetes* and *Firmicutes*) fermentation in the intestine, which in turn could regulate the expression of genes involved in the proliferation, differentiation, and apoptosis of intestinal epithelial cells and affect the composition of the gut microbiota and host inflammatory response (65). Various studies have reported decreased SCFA production or decreased SCFA-producing bacteria (*Roseburia hominis* and *Faecalibacterium prausnitzii*) in IBD-affected patients (64, 65, 120, 121), suggesting a role for SCFAs in inflammatory responses in IBD. The beneficial therapeutic effects of SCFAs have also been observed following the administration of SCFAs or prebiotics in animal models or UC-affected patients (122–126). The effect of SCFAs on IBD is predominantly mediated by regulating both the innate immune response and adaptive immune response (127). In addition, several metabolites produced by intestinal bacteria have been identified to have the ability to affect host metabolism and immunity in experimental colitis or IBD patients (128). The aryl hydrocarbon receptor (AhR) is a transcription factor that resides in the cytoplasm of the intestinal epithelium, macrophages, B cells, T cells, and dendritic cells (129). Kynurenine, a metabolic product derived from the essential amino acid tryptophan, has been identified as an endogenous AhR ligand (130) that regulates the expansion of intraepithelial lymphocytes, innate lymphoid cells, and immune and inflammatory reactions, and maintains normal mucosal function in the gut (131). In the GI tract, diet-derived AhR ligands promote local IL-22 production, which in turn stimulates the production of antimicrobial peptides and mucin (132), thus conferring pathogen resistance and mucosal protection (132). CARD9 null mice have been reported to have impaired immune responses to *Citrobacter rodentium* as shown by the decreased production of colonic IL-6 and IL-17A as well as fewer IL-22-producing innate lymphoid cells in the colon lamina propria (133). In a recent study, Lamas et al. showed that microbiota dysfunction and susceptibility to IBD in CARD9 knockout mice are mainly attributed to their inability to metabolize tryptophan into metabolites that act as AhR ligands. By contrast, the addition of tryptophan-metabolizing *Lactobacillus* strains can attenuate intestinal inflammation in CARD9 null mice (96). These data suggest that tryptophan metabolites are bioactive mediators that regulate the crosstalk between the host immune response and intestinal microbiota ecosystem (134).

#### Stress

Stress is thought to be another risk factor in the development of IBD (135). Data from preclinical and clinical studies have revealed that stress reduction is associated with decreased relapse in patients with UC or CD (136, 137). This effect of stress on IBD is mainly mediated by corticotropin-releasing factor (CRF) signaling (138). In response to stress, CRF is synthesized and released from multiple brain regions including the paraventricular nucleus and hypothalamus. The released CRF stimulates the production of adrenocorticotropic hormone from the pituitary gland, which is transported to the adrenal cortex to induce the synthesis and secretion of cortisol in response to stress (139). CRF signaling can act in peripheral tissues, including the stomach, pancreas, small intestine, and lymphocytes (139–142). Our study and others have shown that CRF signaling can be activated by stress in the intestines of rodents and pigs (143, 144). Importantly, the activation of corticotropin-releasing hormone has been reported to be associated with inflammation of the colonic mucosa and increased intestinal permeability in patients with CD or UC (145–147), thus suggesting the involvement of stress in the pathophysiology of IBD through several mechanisms. First, CRF activation can lead to TNF-α release and protease secretion (hallmarks of IBD) from mast cells, which in turn act on epithelial cells and result in bacterial translocation and over-activation of the immune response due to increased permeability (11, 41, 148). Second, stress-induced HPA activation, alterations in neurotransmitters, and immune function activation can modulate the intestinal microbiota composition and metabolism as well as permeability (149–151). Considering the effect of stress-induced microbiota dysfunction in the pathophysiology of IBD, CRF antagonistic or anti-TNF-α agents have been found to result in favorable outcomes in IBD patients (152). In our recent study, we demonstrated that glutamine supplementation attenuates the stress-induced downregulation of tight junction proteins, increase in permeability, and release of CRF in intestinal tissues (144, 153). Considering the essential role of tight junction proteins in the maintenance of the intestinal barrier and prevention of bacterial translocation, glutamine might be a promising adjuvant in IBD therapeutics. Taken together, environmental factors are emerging as critical contributors to the development of IBD through complex interactions between intestinal bacteria and the host. In addition to dietary factors, the intake of drugs and antibiotics, age, and other environmental factors can influence the intestinal microbiota by interacting with commensal microorganisms (154–157).

## Potential Therapeutic Exploitation by Targeting Intestinal Microbiota

It is increasingly evident that a delicate balance between the gut microbiota and intestinal immune system is required to protect against pathogenic bacteria and contribute to intestinal homeostasis and functions (84). Recent studies in both animal models and clinical patients have highlighted the critical role of the intestinal microbiota in initiating, maintaining, and determining the severity of IBD (72). Restoration of the diversity and composition of the commensal microbiota is emerging as a novel therapeutic intervention for microbial imbalance involved GI diseases, including IBD and IBS (72). Manipulation of the gut microbiota can be achieved by antibiotics, fecal microbiota transplantation (FMT), or probiotics.

### Antibiotics

The rationale for antibiotic therapy in IBD is based on evidence showing that intestinal microbes, including luminal bacteria, have an important role in the pathogenesis of IBD (27, 73, 158). Antibiotic treatment can decrease the abundance of pathogenic bacteria to favor the growth of beneficial bacteria (159, 160). Although antibiotics are commonly used in clinical practice to improve the life quality of patients, their benefits have not been well-established in carefully designed clinical trials with patients affected with IBD (159, 161). Moreover, antibiotic treatment has also been reported to have adverse outcomes due to currently unknown reasons or lead to various side effects in fetuses and children affected by IBD (162). A meta-analysis has demonstrated that long-term exposure to antibiotics is associated with a higher incidence of CD because of their interfering effect on the intestinal flora (163). Moreover, antibiotic intervention early in life might influence the development and maturation of the intestinal immune system of the host (164). Another concern regarding antibiotic treatment for IBD is the development and spread of bacterial resistance to antibiotics as well as the rebound of intestinal bacteria after the cessation of therapy (162). Although antibiotics are potent in regulating microbiome ecological diversity, these issues should be carefully addressed for IBD treatment in the future.

### Fecal Microbiota Transplantation

Fecal microbiota transplantation, also known as “fecal bacteriotherapy” or “fecal infusion,” is the transfer of intestinal bacteria from a healthy donor to restore the intestinal microbiota of a diseased individual (165, 166). FMT has been adapted in clinical practice for CDI, which cannot be eliminated with antibiotics alone, and has been proven to be more effective than antibiotic treatment (166, 167). Thereafter, FMT has been evaluated in several microbiota-driven diseases, including IBD (168, 169) and metabolic syndrome (170), and has gained interest as a novel therapy option. In contrast to the impressive results of treatment for CDI (92%) (171), FMT has been reported to reduce symptoms in about 20% of IBD patients (172). This huge difference might be attributed to several reasons. First, the severity and duration of symptoms in clinical trials may influence the outcome; thus, positive outcomes are observed only in some patients enrolled in the clinical trials (173). Second, IBD is a chronic disease associated with various factors, such as gut microbiota impairment and genetic and/or environmental factors; thus, the development of IBD is a synergetic effect of multiple factors instead of a single one (4, 169). Third, the immune system of IBD patients is upregulated due to chronic inflammation (174), which might affect the therapeutic effect of FMT. Fourth, the composition of the gut microbiota from donors is different (175) and might affect the clinical outcome. Fifth, the FMT protocol used in each trial (including the criterion for donor selection, patient preparation, the number and composition of bacteria infused, and route of administration) is different for each patient (172), which will result in different therapeutic effects. All these factors could contribute to a lower clinical outcome compared with that of CDI. Nevertheless, it is too early to draw conclusions on the potential of FMT therapy. Additional clinical studies using a standard protocol are required to evaluate its efficacy and safety before prescribing it for IBD patients (173).

### Probiotics

Probiotics are living microorganisms that exert beneficial effects on the host by modulating the intestinal microflora (176, 177). *Lactobacillus* and *Bifidobacterium* species, two of the most common probiotic bacteria, have been found not only to improve immune system responses but also to exert a positive effect on the preexisting microflora stability, to inhibit pathogen colonization, and to enhance mucosal trophic effects by stimulating intestinal epithelial cell barrier responses (178, 179). Tao et al. showed that the probiotic *Lactobacillus* GG releases soluble factors, which in turn stimulate the synthesis of heat-shock proteins through the p38 MAPK pathway, thus exerting a cytoprotective effect on intestinal epithelial cells (180). In a randomized study, 39 UC patients were treated with *L. rhamnosus* GG daily, while the remaining patients (*N* = 78) were not treated; the progression of UC was inhibited by probiotic administration, demonstrating the significant clinical benefit of probiotics (181). In another study, VSL#3, a high-potency probiotic medical food containing eight different strains, could induce remission and prevent the relapse of inflammatory disease in patients with mild to moderately active UC (182–184). Other probiotics, such as *Bifidobacterium bifidum, L. acidophilus* (185), and *L. reuteri ATCC* 55730 (186), have also been reported to be associated with beneficial effects in IBD patients.

**Figure 3:**
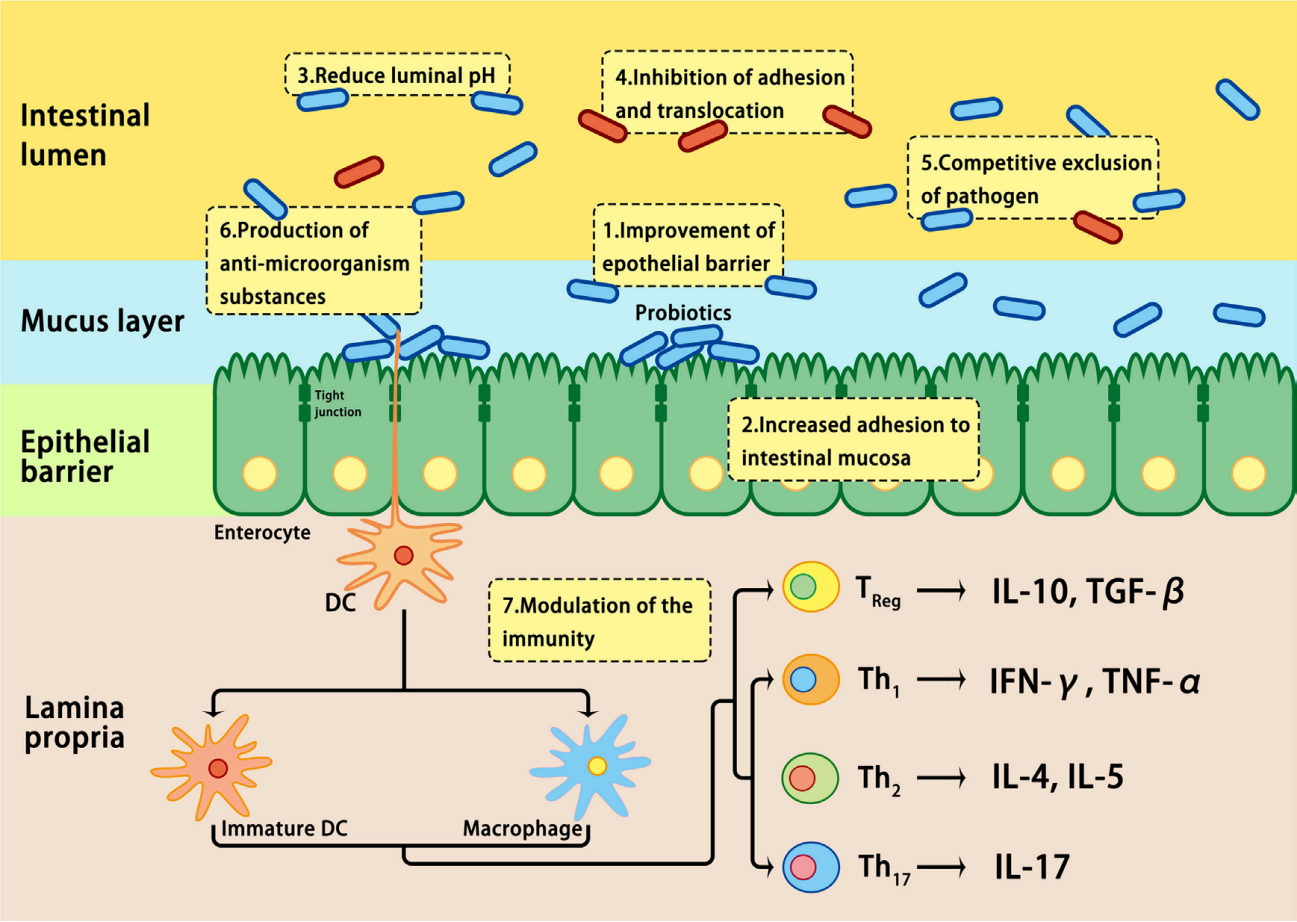
Mechanisms involved in probiotic-induced protection against intestinal dysbiosis. Probiotics suppress pathogens through various actions, including lowering luminal pH, production of antimicrobial proteins, inhibition of adhesion and translocation of flora, competitive exclusion of pathogens, improvement of epithelial barrier, enhancement of adhesion of commensal bacteria to the intestinal mucosa, and modulation of gastrointestinal mucosal immune system.

The effects of probiotics are mediated by modulating the mucosal inflammation system, enhancing competitive exclusion to pathogenic bacteria, regulating the secretion of cytokines involved in the development of IBD, or modulating intestinal permeability (109, 178) (Figure 3). It is well known that probiotics express pathogen-associated molecular patterns; they can mimic the function of commensal bacteria by engaging and/or activating the pattern recognition receptors on epithelial mucosal surfaces, thus regulating the expression of genes involved in the host immune response (178). In addition, the beneficial effects of probiotics have been attributed to the restoration of the number and function of goblet cells and the stimulation of the mucosal immune system to secrete protective immunoglobulins, such as secretory IgA, protective defensins, and bacteriocidins in the intestinal tract (187). Although the practical application of probiotics has been encouraged by positive results in clinical trials involving UC patients, clinical trials involving CD patients have not inspired much enthusiasm (178, 188). Further studies on the efficacy, safety, and underlying mechanisms of action are required before probiotics may be recommended for the treatment of IBD patients (188).

## Perspective

The role of the interaction between the intestinal microbiota and host immune response in the pathogenesis of IBD has attracted much attention because of the application of next-generation sequencing technology and the availability of genetically engineered animal models. Intestinal dysbiosis is emerging as a risk factor because of its functional role in the maintenance of intestinal homeostasis, activation of the immune response of the intestinal epithelium, and its crosstalk with other factors through genetic or epigenetic mechanisms. Based on advances in our understanding of the microbiota in IBD pathogenesis, several therapeutic interventions have been investigated for restoring the commensal microecology, and some of them have shown impressive results in clinical trials or experimental animal models. However, various questions need to be addressed. The intestinal microbiota is subjected to changes in both host and exogenous factors, and it is largely unknown how dysbiosis is triggered and leads to chronic inflammation. Furthermore, an imbalance in bacterial populations is associated with various diseases; the pathogenic implications of specific microbes in CD or UC, as well as underlying mechanisms, remain to be determined. Despite the occurrence of dysbiosis in patients with CD and UC, it is unclear whether alterations in the intestinal flora contribute to the development of IBD or are instead a consequence of this disorder. The treatment of dysbiosis through FMT or prebiotic administration has produced favorable results in clinical trials involving IBD patients; however, both their safety and efficacy have to be determined before they can be considered as a therapeutic strategy. Additional studies on the interplay between the microbiota and intestinal epithelium are of great importance to advance our understanding of the role of the microbiota in the pathogenesis of IBD and to identify potential therapeutic strategies by manipulating the intestinal microbiota.

## Author Contributions

YY and ZW designed the study; MZ and YW contributed to the literature search; KS, YW, and PT generated the figures and discussed on the revision; ZW revised and finalized the manuscript.

## Conflict of Interest Statement

The authors declare that the research was conducted in the absence of any commercial or financial relationships that could be construed as a potential conflict of interest.
